# The Concentration and Duration of Lipopolysaccharide Stimulation Produce Different Cytokine Responses in an Ex Vivo Whole Blood Model in Horses

**DOI:** 10.3390/vetsci12111090

**Published:** 2025-11-16

**Authors:** Natalie Mitlyng, Kallie J. Hobbs, Bethanie L. Cooper, M. Katie Sheats

**Affiliations:** 1 Department of Clinical Sciences, North Carolina State University, Raleigh, NC 27695, USA; nmmitlyn@ncsu.edu (N.M.); bplewis2@ncsu.edu (B.L.C.); 2 Department of Clinical Sciences, Texas A&M University, College Station, TX 77845, USA

**Keywords:** sepsis, endotoxemia, systemic inflammatory response syndrome

## Abstract

Lipopolysaccharide (LPS) is frequently used in equine research to model clinical endotoxemia; however, there is no standardized protocol for inducing cytokine production in equine whole blood. To address this knowledge gap, the goal of this study was to compare the cytokine responses elicited by three different LPS stimulation protocols. Whole blood was collected from six healthy horses (aged 5–30 years; mixed breeds and genders) from the North Carolina State University teaching herd (IACUC #23-412). Sixty milliliters of heparinized blood were aseptically drawn and divided into 15 mL aliquots. Samples were stimulated with LPS at concentrations of 100 ng/mL, 1000 ng/mL, or using a two-hit model (500 ng/mL initially and again at 1.5 h). Incubation occurred at 37 °C on an orbital shaker for time points ranging from 1.5 h to 24 h. Cytokine concentrations were measured using the Cornell Equine Cytokine and Chemokine Panel and were compared to non-stimulated controls. LPS stimulation induced the production of TNF-α, IL-1β, IL-10, CCL5, and CCL11 in a time- and concentration-dependent manner. Notably, reliable and robust cytokine responses were observed only after 12 h of stimulation with either 1000 ng/mL or the two-hit 500/500 ng/mL protocol. These findings suggest that both the concentration and duration of LPS exposure significantly influence cytokine expression in equine whole blood. Therefore, the optimal stimulation protocol may vary depending on the specific cytokine of interest, and careful consideration of these variables is essential for designing reproducible and physiologically relevant ex vivo models of equine endotoxemia.

## Simple Summary

Numerous studies have used lipopolysaccharide (LPS) to simulate endotoxemia in horses in vivo, but standardized methods for inducing cytokine production in whole blood ex vivo are lacking. This study compares cytokine response to three different LPS stimulation protocols using blood from six healthy horses. Blood samples were exposed to different LPS concentrations (100 ng/mL, 1000 ng/mL, or a two-step 500/500 ng/mL protocol) and incubated for up to 24 h. Cytokine levels were measured and compared to controls. Results show that significant cytokine responses—especially for TNF-α, IL-1β, IL-10, CCL5, and CCL11—occurred reliably only after 12 h of stimulation with either 1000 ng/mL or the two-step protocol. These findings emphasize the importance of both LPS concentration and exposure duration in designing effective ex vivo models of equine endotoxemia.

## 1. Introduction

Sepsis in horses is currently defined as a dysregulated host-systemic inflammatory response to infection [1]. Endotoxemia in horses refers to a severe systemic inflammatory response caused by the suspected presence of lipopolysaccharide (LPS) in the bloodstream. The dysregulated immune response in these conditions is often referred to as a cytokine storm [2]. Manipulation of this cytokine storm is a primary focus in the literature relating to systemic inflammatory disorders. In humans, a widely accepted method of studying sepsis-relevant cytokines is the ex vivo stimulation of whole blood using Lipopolysaccharide (LPS). Although the use of LPS infusion for in vivo studies is common in equine research, potential side effects include fever, depression, and mild colic. An alternative option to in vivo studies is an ex vivo whole blood model; however, there is a paucity of literature on the use of LPS for whole-blood stimulation in the horse. The advantage of the ex vivo LPS whole blood model is that it allows for cytokine manipulation without the animal health risks associated with in vivo studies. In patients with clinical endotoxemia, laminitis and colic are common secondary complications [3], in addition to cardiovascular compromise, coagulopathy, and multiple organ failure. Although there are many cytokines involved in the immune response during endotoxemia, most of the literature focuses on the measurement and roles of TNF- α, IL-1β, IL-6, IL-10, and IFN-γ [4,5,6].

In the limited literature available, there are reports on the use of leukocyte-rich plasma (LRP) [7] as an ex vivo model for endotoxemia. To our knowledge, there are no studies describing LPS-induced cytokines in equine whole blood. In LRP models, the peak production of endotoxemia-relevant cytokines occurred at six hours, with cytokine levels decreasing by 24 h. For in vivo models, the time taken for cytokine production is frequently influenced by the specific target cytokine. TNF- α and IL-1β have been noted in the literature [8] to spike at one hour and then again at six hours. These distinct temporal responses may suggest that the immune response undergoes a two-pronged response. This suggests that, under ex vivo conditions, a two-hit model may better mimic the pathophysiology of the immune response in the horse.

Given this, the objective of this study was to compare the cytokine response of TNF-α, IL-1β, IL-10, and IFN-γ using three different LPS stimulation protocols in an ex vivo whole-blood model in the horse. These findings will help to inform investigators utilizing LPS to stimulate cytokine production in equine whole blood ex vivo.

## 2. Materials and Methods

### 2.1. Animals

Six healthy horses from the North Carolina State University College of Veterinary Medicine teaching herd were used for the purpose of this study. The ages of the horses varied from 5 to 30 years. Of the 6 horses, 3 were geldings and 3 were mares. All of the horses were kept on a pasture in similar conditions. Body condition score (BCS) was assessed for each horse. Inclusion criteria were clinically healthy horses with no known history of systemic inflammatory disorders and no medications administered for at least two weeks prior to sampling. Horses showing clinical illness or extreme BCS (<3 or >8) were excluded to avoid confounding cytokine responses. All procedures were approved by the NCSU Institutional Animal Care and Use Committee (IACUC 23-110).

### 2.2. Blood Sampling

Sixty milliliters of whole blood were obtained aseptically using an 18 g needle and heparinized syringe. Each stimulation condition was prepared in separate 15 mL conical polypropylene tubes, and all experiments were performed in the same batch per horse to avoid inter-batch variability.

### 2.3. LPS Stimulation and Cytokine Analysis

Whole blood was stimulated with LPS from E. coli 055:B5 (Sigma Aldrich, St. Louis, MO, USA) at 0 ng/mL, 100 ng/mL, 1000 ng/mL, or by a two-stimulation method with a 500 ng/mL initial concentration, followed 90 min later by another 500 ng/mL stimulation. Blood was placed in an orbital shaker at 37 C while undergoing stimulation. Plasma was immediately separated off and frozen within 15 min of collection at 1.5 h, 6 h, 12 h, and 24 h for cytokine protein analysis. The LPS protocol follows modified methods from prior studies in leukocyte rich plasma and human whole blood, adapted for equine whole blood ex vivo [7,9]. Plasma samples were analyzed using the Equine Cytokine 5-plex and Equine Chemokine 6-plex kits (Animal Health Diagnostic Center, Cornell University, Ithaca, NY, USA). The kits rely on Luminex^®^ bead-based multiplex immunoassay technology, which uses color-coded fluorescent microspheres conjugated with monoclonal antibodies for the capture and detection of specific cytokines and chemokines. This technology enables the simultaneous measurement of multiple analytes from a single sample, increasing sensitivity and efficiency compared to traditional assays [10]. Cytokines analyzed included TNF-α, IL-1β, IL-10, IFN-γ, IL-4, IL-17, and IFN-α. Chemokines analyzed included CCL2, CCL3, CCL5, and CCL11.

### 2.4. Statistics

All data were analyzed using GraphPad Prism (Version 10). For each horse, control non-stimulated blood samples were compared to stimulated samples at corresponding time points. Time points showing a statistically significant difference between the control and non-stimulated samples were excluded from further analysis. The normality of the data distribution was assessed using the Shapiro–Wilk test. Statistical comparisons were performed using one-way ANOVA for normally distributed data and Kruskal–Wallis tests for non-normally distributed data. Cytokines that did not demonstrate a significant difference between the control and stimulated samples at any time point were excluded from further analysis. Fold change in cytokine concentrations was calculated for each time point and visualized graphically to assess the trends over time (Supplemental Tables S1 and S2).

## 3. Results

### 3.1. LPS Stimulates the Production of TNF-a, IL-1β and IL-10

**Figure 1:**
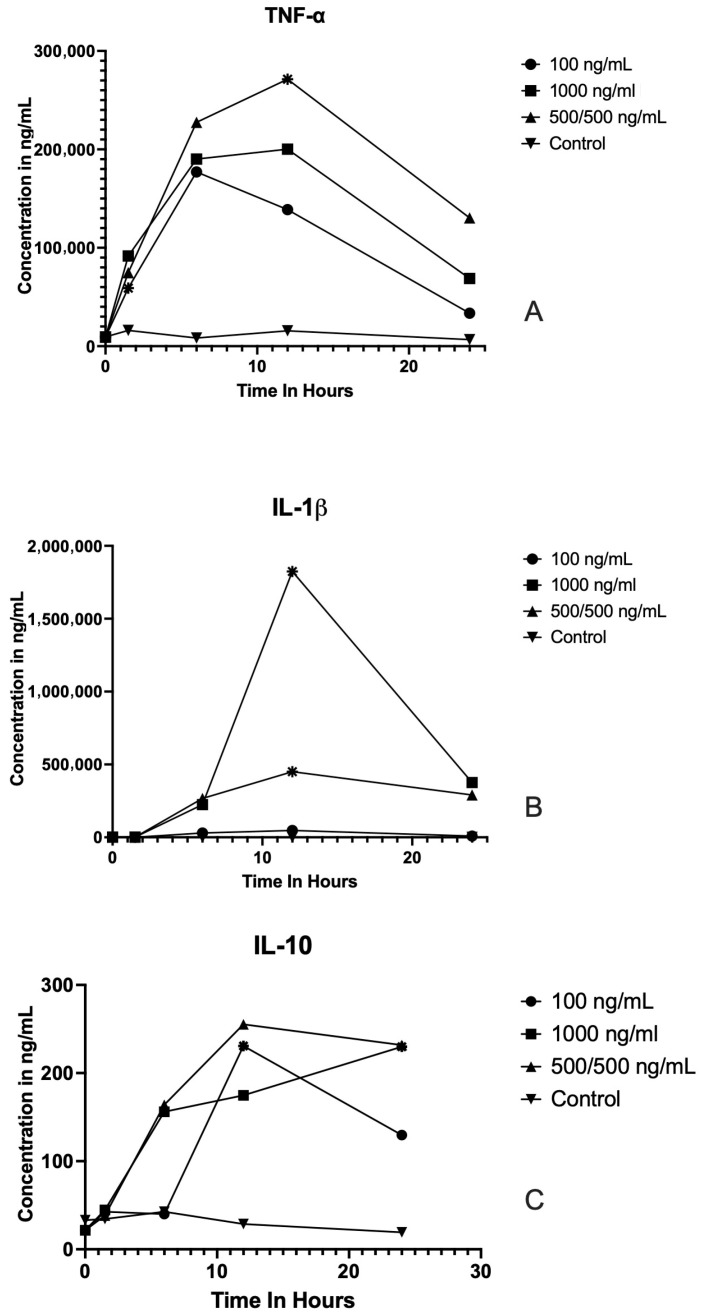
Cytokine concentration profiles following LPS stimulation at different concentrations and time points. Line graphs show the concentrations (ng/mL) of TNF-α (**A**), IL-1β (**B**), and IL-10 (**C**) measured at baseline and 1.5, 6, 12, and 24 h post LPS stimulation in cells treated with LPS at 0 ng/mL (control), 100 ng/mL,1000 ng/mL, or 500/500 ng/mL. Each point represents the mean. Significant differences (*p* < 0.05) compared to baseline or between indicated time points are denoted by asterisks. Cytokine concentrations peaked within the first 6–12 h following stimulation and generally declined by 24 h. TNF-α and IL-1β displayed more robust responses than IL-10, with higher LPS concentrations yielding greater cytokine release.

No significant differences were observed for TNF-α, IL-1beta, or IL-10 levels between the initial starting baseline and the control at each time point (Figure 1 and Supplemental Table S1). LPS stimulation induced time-dependent changes in the secretion of TNF-α, IL-1β, and IL-10 in samples treated with three different concentrations (100 ng/mL, 1000 ng/mL, and 500/500 ng/mL), as shown in Figure 1. 

**TNF-α:** Across all concentrations, TNF-α secretion showed a marked increase following LPS stimulation. At 100 ng/mL, TNF-α levels were significantly higher at 6 h post-stimulation compared to baseline (*p* = 0.04). The greatest TNF-α response was observed at 1000 ng/mL, where concentrations peaked at 12 h after LPS exposure (*p* = 0.04). In the 500/500 ng/mL group, TNF-α concentrations peaked at 12 h post-stimulation (*p* = 0.02), before declining at 24 h. **IL-1β:** For the 100 ng/mL condition IL-1β concentration, there was no statistical difference at any time point. At the higher concentration of 1000 ng/mL, IL-1β release was significantly elevated at 12 h compared to baseline (*p* = 0.03), followed by a rapid decline by 24 h. In the 500/500 ng/mL group, IL-1β levels peaked at 12 h (*p* = 0.02). **IL-10:** LPS stimulation also resulted in enhanced IL-10 secretion, although levels were substantially lower relative to TNF-α and IL-1β. At 100 ng/mL, IL-10 showed no statistical difference from baseline at any time point. At 1000 ng/mL, IL-10 concentrations were significantly higher at 24 h compared to baseline (*p* = 0.03). In the 500/500 ng/mL group, IL-10 secretion was significantly elevated relative to baseline at 12 h (*p* = 0.02).

### 3.2. LPS Does Not Stimulate the Production of IFN-α, IL-4, IL-17 or IFN-γ, CCL2 or CCL3

No significant differences were observed between the initial starting baseline and the control at each time point for IFN-a, IL-4, IL-17, IFN-g, CCL2, or CCL3. There was no significant difference between the baseline and stimulation for any tested LPS concentration or time point. The exposure of whole blood to all three stimulant concentrations failed to elicit cytokine production at any evaluated time point.

### 3.3. LPS Stimulates the Production of Chemokines CCL5 and CCL 11 at All Concentrations and Time Points

**Figure 2:**
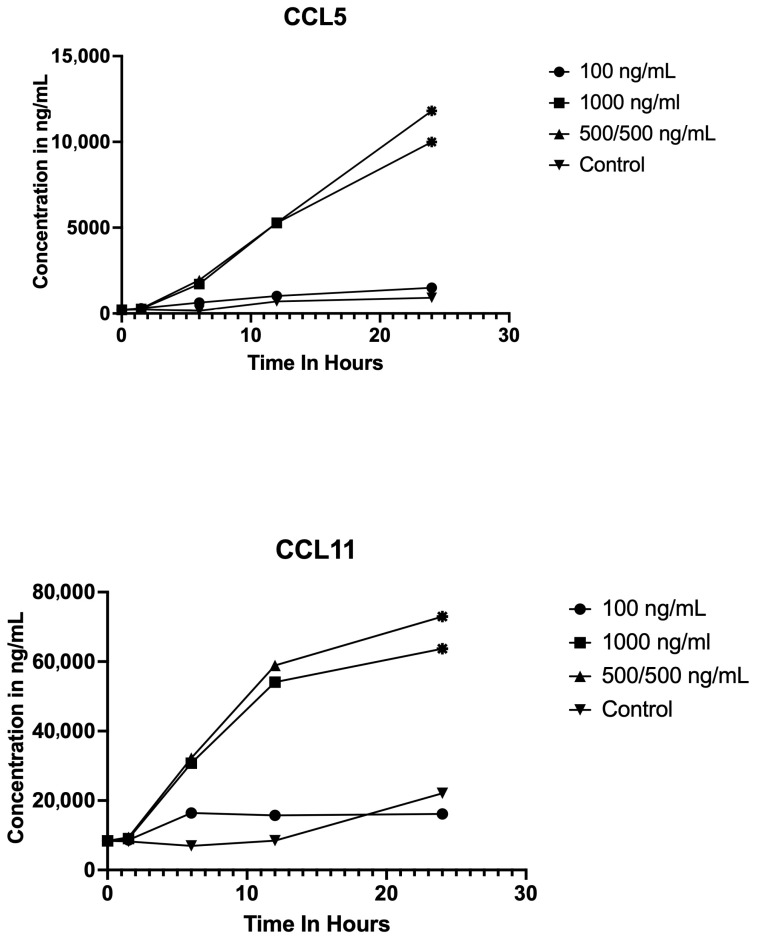
Chemokine concentration profiles following LPS stimulation at different concentrations and time points. Line graphs display the concentrations (ng/mL) of CCL5 (**top**) and CCL11 (**bottom**) measured at baseline and at 1.5, 6, 12, and 24 h post LPS stimulation in samples treated with 0 ng/mL (control), 100 ng/mL, 1000 ng/mL, and 500/500 ng/mL LPS. Each point represents the mean. Statistically significant differences (*p* < 0.05, *p* < 0.01) compared to baseline denoted by asterisks at the points. CCL5 and CCL11 concentrations increased over time after LPS stimulation, with peak concentrations observed at the 24 h time point, especially at higher LPS doses.

LPS stimulation produced distinct time-dependent increases in the chemokines CCL5 and CCL11 at the 1000 ng/mL and 500/500 ng/mL concentrations, as depicted in Figure 2. CCL5 and CCL11 concentrations exhibited no significant difference at any time point when treated with 100 ng/mL. At both 1000 ng/mL and 500/500 ng/mL doses, CCL5 levels showed a significant elevation at 24 h post-stimulation (*p* < 0.01 for both conditions). When treated with 1000 ng/mL or 500/500 ng/mL, LPS displayed a significant increase in CCL11 concentrations at 24 h relative to baseline (*p* < 0.05 for both conditions).

## 4. Discussion

In this study, we measured cytokine production in equine whole blood in response to three different LPS stimulation protocols. Our results show that stimulation with 1000 ng/mL LPS, or the “two-hit” approach with 500 ng/mL of LPS followed 90 min later with 500 ng/mL LPS followed by 12 to 24 h incubation, produced the highest concentrations of cytokines in equine whole blood. Our results also show that the appropriate LPS stimulation protocol may vary depending on the cytokine of interest. There were no significant differences between baseline and control time points, indicating that any changes seen after stimulation can reasonably be attributed to the effects of LPS on cytokine production pathways.

Notably, TNF-α, IL-1β, IL-10, CCL5, and CCL11 exhibited robust production following LPS stimulation, with distinct patterns observed across different concentrations and time points. For chemokines CCL5 and CCL11, the LPS model was able to create reliable induction across all time points only at high LPS concentrations, suggesting that further exploration of whole blood LPS stimulation for a chemokine model may be warranted.

Baseline and control cytokine and chemokine concentrations demonstrated no statistically significant differences and exhibited minimal variation across all measured time points. This consistency indicates that the observed increases in cytokine production were not attributable to inherent fluctuations over time. Instead, these findings strongly support lipopolysaccharide (LPS) stimulation as the primary driver of the elevated cytokine and chemokine levels detected in the experimental samples.

TNF-α and IL-1β levels increased significantly across all tested LPS concentrations, peaking at 12 and 24 h post-stimulation with 1000 ng/mL of LPS. This aligns with previous in vitro studies demonstrating peak TNF-α production in whole-blood cell culture supernatants 12 h after inoculation with 1000 ng/mL of LPS [11]. However, this contrasts with in vivo equine studies, which reported peak TNF-α and IL-1β gene expression and concentration within 60 min of LPS administration, with levels diminishing by 3 h [6]. This discrepancy between in vitro and in vivo studies likely results from rapid LPS clearance by the liver and spleen in vivo, which occurs within 2–4 minutes of administration [12].

Similarly to TNF-α and IL-1β, IL-10 showed a significant fold change increase when stimulated with 1000 ng/mL of LPS at the 12–24 h time point. While limited literature exists on ex vivo whole blood models and IL-10 concentrations in horses, studies in other species have reported peak concentrations at 5 and 12 h [13,14]. This contrasts with in vivo IL-10 responses, which typically peak around 8 h. This later peak in vivo reflects IL-10’s immunosuppressive role in downregulating excessive inflammatory cytokine responses [15]. This difference in timing between IL-10 production and that of TNF-α and IL-1β (which are directly triggered by LPS) likely stems from IL-10 production being dependent on the prior production of other cytokines, rather than a direct result of LPS-stimulation [16,17].

Although not the primary focus of this study, several chemokines were analyzed. Statistically significant differences between baseline and control time points for CCL2 and CCL3 across all horses precluded determining whether LPS stimulation or environmental factors caused the observed increases in these chemokines. In contrast, stimulation of CCL5 and CCL11 production appeared reliable. While environmental variation cannot be entirely excluded, the established role of LPS induced CCL5 and CCL11 expression [18] suggests that LPS stimulation was the likely cause of the increased concentrations. It is important to note that the baseline concentrations of CCL5 and CCL11 in the horses selected for this study were at the upper end of the normal reference range for these chemokines in horses. Baseline variation is a recognized limitation in equine studies, as individual horses can exhibit differences within the normal range [19]. However, these concentrations remained relatively stable in the control samples throughout the study period, which supports that the observed increases were attributable to LPS stimulation rather than inherent variability in baseline values.

In contrast, IFN-α, IL-4, IL-17, and IFN-γ did not exhibit significant increases in response to any LPS concentration at the measured time points. This suggests that our current model may not adequately stimulate these cytokines, or that further optimization is required to elicit their production effectively. The lack of response from IFN- α, IL-17, and IL-4 was not completely unexpected, given the previously established evidence linking IFN-α to viral insult, IL-17 to autoimmune disease pathways, and IL-4 to allergic response. Similar findings have been reported in other models. For instance, a study using human PBMCs found that probiotic stimulation failed to induce significant production of IL-4 and IFN-γ [20], suggesting that LPS alone may not trigger these immune pathways, or that additional immune stimuli are required. Another study examining human PBMCs found that E. coli LPS failed to induce significant IFN-γ production, likely due to the regulatory effects of IL-10, which inhibited the IFN-γ response [21]. A study in response to *Neisseria gonorrhoeae* infection also found a broad cytokine profile, including both Th1 and Th2 cytokines, but a weaker IFN-γ response to *E. coli* LPS. This study highlighted how immune responses are cross-regulated by IL-10, suggesting that regulatory factors might suppress IFN-γ production, potentially explaining the absence of a robust IFN-γ response in our model [22]. These findings suggest that similar regulatory mechanisms may be at play in horses, underscoring the need for further optimization of LPS stimulation protocols.

This study has several limitations. While the cytokines targeted are relevant to the pathophysiology of endotoxemia, our list was limited to the cytokines included in the assay. To maintain assay consistency, we opted not to include other methods to measure additional cytokines in this study. Another limitation was the lack of hourly cytokine data, which was due to budget limitations. Lastly, this study did not investigate the 48 and 72 h time points, which have been reported in other studies. These time points were omitted because previous studies consistently showed a significant decline in cytokine levels at these later time points.

## 5. Conclusions

This study offers new data regarding LPS-stimulated cytokines in equine whole blood ex vivo. The findings suggest that LPS effectively induces TNF-α, IL-1β, IL-10, CCL5, and CCL11 production in a time- and concentration-dependent manner. Among the tested conditions, 1000 ng/mL of LPS for 12 h yielded the most significant cytokine production. Additionally, the majority of cytokines had decreased by 24 h. Overall, these findings contribute to the ongoing optimization of models of equine sepsis and endotoxemia, offering a foundation for further investigations into cytokine dynamics and therapeutic interventions in equine inflammatory conditions.

## Data Availability

The original contributions presented in this study are included in the article/Supplementary Materials. Further inquiries can be directed to the corresponding authors.

## Supplementary Materials

The following supporting information can be downloaded at https://www.mdpi.com/article/10.3390/vetsci12111090/s1. Table S1: Fold change in TNF-α, IL-1β, and IL-10 expression following LPS stimulation at various time points; Table S2: Fold change in CCL5 and CCL11 expression following LPS stimulation at various time points.

## Abbreviations

The following abbreviations are used in this manuscript:

LPS	Lipopolysaccharide
TNF-a	Tumor Necrosis Factor Alpha
IL-1b	Interleukin-1 beta
IL-10	Interleukin 10
CCL5	C-C Motif Chemokine Ligand 5
CCL11 C-C	Motif Chemokine Ligand 11
BCS	Body Condition Score
LRP	Leukocyte Rich Plasma
