# Supplementary material for: The Concentration and Duration of Lipopolysaccharide Stimulation Produce Different Cytokine Responses in an Ex Vivo Whole Blood Model in Horses
Source: Vet Sci. 2025 Nov 16;12(11):1090. doi: 10.3390/vetsci12111090 (PMC12656798; doi:10.3390/vetsci12111090)
Supplement: Supplementary file 1 [file vetsci-12-01090-s001.zip › vetsci-3952124-supplementary.pdf]

Cytokine	Time	LPS Stimulation Concentration			Control
		100 ng / mL	1000 ng / mL	500 / 500 ng / mL	0 ng/mL
TNF-α	90 minutes	5.49 ± 5.55	8.26± 10.18	6.79±+7.98	0.79±0.98
	6 hours	31.66 ± 30.15	49.54±51.62	353.6±565.95	1.68±2.20
	12 hours	13.47 ± 8.62	234.1±366.3	960.93±1619.76	1.64±2.24
	24 hours	5.53 ± 4.87	266.95±440.25	16.12±4.25	0.65±0.71
IL-1β	90 minutes	1±1	1±1	1±1	0.39±0.68
	6 hours	491±503.02	3686±1190.66	4272±1919.76	0±0
	12 hours	142±96.77	6691±1865.33	7378±4981.24	0±0
	24 hours	179±132.6	6161±2281.44	4771±3320.45	0±0
IL-10	90 minutes	1.5±23.86	4.36±0.05	1.07±0.13	0.98±0.03
	60 hours	1±0	5.89±4.58	6.00±3.27	1.1±0.36
	12 hours	5.9±14.72	9.58±8.36	6±6.36	1.26±1.04
	24 hours	3.75±125.9	10.25±6.7	10.3±0.7	0.67±0.69

Supplemental Table S1: Fold change in TNF-α, IL-1β, and IL-10 expression following LPS stimulation at various time points. Data represent the mean ± standard deviation (SD) for each cytokine measured at 90 minutes, 6 hours, 12 hours, and 24 hours after stimulation with 100 ng/mL, 1000 ng/mL, and a 500/500 ng/mL two-hit model of LPS. No significant statistical difference was observed between baseline and control at any time point analyzed for TNF-α, IL-

1β, or IL-10. Results are expressed as fold change relative to baseline values. A value of 0 designates that the concentration was below the threshold of the assay.

Chemokine	Time	LPS Stimulation Concentration			Control
		100 ng / mL	1000 ng / mL	500 / 500 ng / mL	0 ng/mL
CCL5	90 minutes	1.59 ± 0.37	6.96± 0.37	1.54±0.53	1.34±0.51
	6 hours	4.83 ± 1.87	1.42 ± 0.45	8.06±5.34	4.18±2.64
	12 hours	5.83 ± 2.09	26.37± 27.33	40.83±27.6	4.01±2.11
	24 hours	13.9± 10.15	67.28± 71.58	87.36±74.14	6.8± 7.9
CCL11	90 minutes	1.03±0.19	1.08± 0.7	1.12±0.5	0.98± 0.22
	6 hours	3.03±2.19	1.58±0.31	1.72±0.7	1.1± 0.92
	12 hours	1.89±0.22	2.66±0.94	3.0±1.78	1.4± 0.71
	24 hours	1.82±1.44	3.17±1.06	3.7±2.2	1.1± 0.08

Supplemental Table S2: Fold change in CCL5 and CCL11 expression following LPS stimulation at various time points. Data represent the mean ± standard deviation (SD) for each chemokine measured at 90 minutes, 6 hours, 12 hours, and 24 hours after stimulation with 100 ng/mL, 1000 ng/mL, and a 500/500 ng/mL two-hit model of LPS. No significant statistical difference was observed between baseline and control at any time point analyzed for CCL5 or CCL11. Results are expressed as fold change relative to baseline values.
